# *RET* fusion mutation detected by re-biopsy 7 years after initial cytotoxic chemotherapy: A case report

**DOI:** 10.3389/fonc.2022.1019932

**Published:** 2022-11-14

**Authors:** Kei Morikawa, Hiroshi Handa, Junko Ueno, Hajime Tsuruoka, Takeo Inoue, Naoki Shimada, Junki Koike, Seiji Nakamura, Yoshiharu Sato, Masamichi Mineshita

**Affiliations:** ^1^ Division of Respiratory Diseases, Department of Internal Medicine, St. Marianna University School of Medicine, Kawasaki, Japan; ^2^ Department of Pathological Diagnosis, St. Marianna University School of Medicine, Kawasaki, Japan; ^3^ DNA Chip Research Inc., Tokyo, Japan

**Keywords:** RET mutation detected by re-biopsy case report, gene mutation, re-biopsy, *RET* fusion lung cancer, selpercatinib

## Abstract

Personalized medicine using molecular-targeted drugs to achieve better therapeutic response and long-term prognosis is common practice for lung cancer treatment. However, in cases before gene batch tests were available, medical treatment continued without the detection of rare mutations. We report a sixty-seven-old year man diagnosed with adenocarcinoma T1cN3M1a, stage IVA. Initial screening performed 7 years earlier using EGFR mutation and ALK immunohistochemical tests were negative. Although first-line cytotoxic combination chemotherapy was remarkably effective, a gradual regression of the primary lesion was noted. After a recent bronchoscopic re-biopsy, *RET* fusion was detected by gene panel test. In addition, we were able to confirm *RET* from FFPE specimens obtained from 7-year-old pleural effusion cell blocks. Subsequent administration of the molecular-targeted drug selpercatinib, was highly effective for the primary lesion and all metastatic lesions including brain metastases. We describe a case of *RET* fusion-positive lung cancer where molecular targeted therapy and cytotoxic drug showed a drastic response and long-term therapy was well maintained. Next generation sequencing was able to correctly diagnose *RET* fusion mutation using re-biopsy specimen after going undiagnosed for 7 years.

## Introduction

Personalized medicine for lung cancer patients using molecular-targeted drugs and immune checkpoint inhibitors has become widespread due to their high response rates and long-term prognosis (1–3). To date, epidermal growth factor receptor (*EGFR*) mutation, anaplastic lymphoma kinase (*ALK*) fusion genes, *ROS1*, *BRAF*, *MET* exon 14 skipping mutations, *RET* fusion genes and their corresponding molecular-targeted drugs have been approved by the Food and Drug Administration (FDA). Furthermore, *KRAS* mutation, *EGFR* and *HER2* exon 20 insertion and their corresponding molecular-targeted drugs will soon be available.

However, in cases before gene batch tests were available, the patient’s medical treatment would have progressed without the detection of rare mutations. It is presumed that malignancy and the therapeutic effects of cytotoxic drugs differ depending on the type of gene mutation (4, 5). Therefore, even in cases with mutation-positive advanced lung adenocarcinoma, long-term clinical courses can be maintained without the administration of molecular-targeted drugs. In particular, even in cases where the initial treatment was successful, the initial sample might not be suitable for gene panel testing due to the deterioration of nucleic acid quality over time. Furthermore, it can be difficult to select the same location for re-biopsy since the lesion might have been altered by treatment. Herein, we report a rare case in where *RET* fusion was detectable by gene panel test after re-biopsy seven years after the initial diagnosis. Sequential administration of the molecular-target drug selpercatinib showed a drastic response.

## Case report

**Figure 1:**
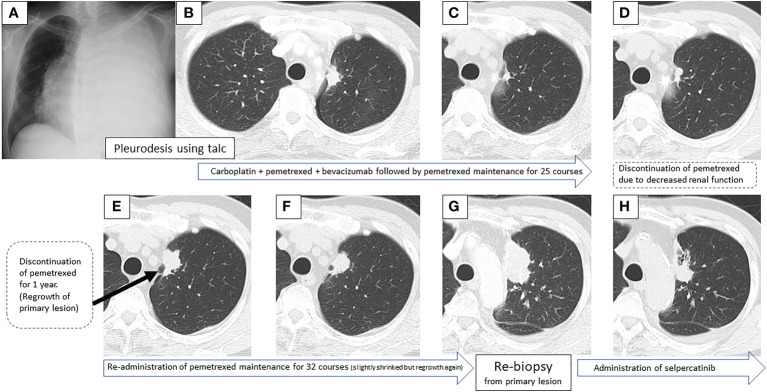
Sequential images of the primary lesion over 7 years, from the time of the first visit to the introduction of the second treatment.

A 67-year-old male, never smoker with no remarkable medical history, was referred to our hospital with a massive left pleural effusion (**Figure 1A**). The cytological evaluation of the left pleural effusion was class V adenocarcinoma, consistent with the cell block pathological assessment. Pleurodesis using talc dilated the lungs well, and a 25 × 17 mm nodule at the left S^1 + 2^a was presumed to be the primary lesion (**Figure 1B**). Clinically, he was diagnosed with adenocarcinoma T1cN3M1a, stage IVA. Initial gene mutation screening seven years earlier using Cobas^®^ EGFR mutation and ALK immunohistochemical (IHC) tests were negative.

**Figure 2:**
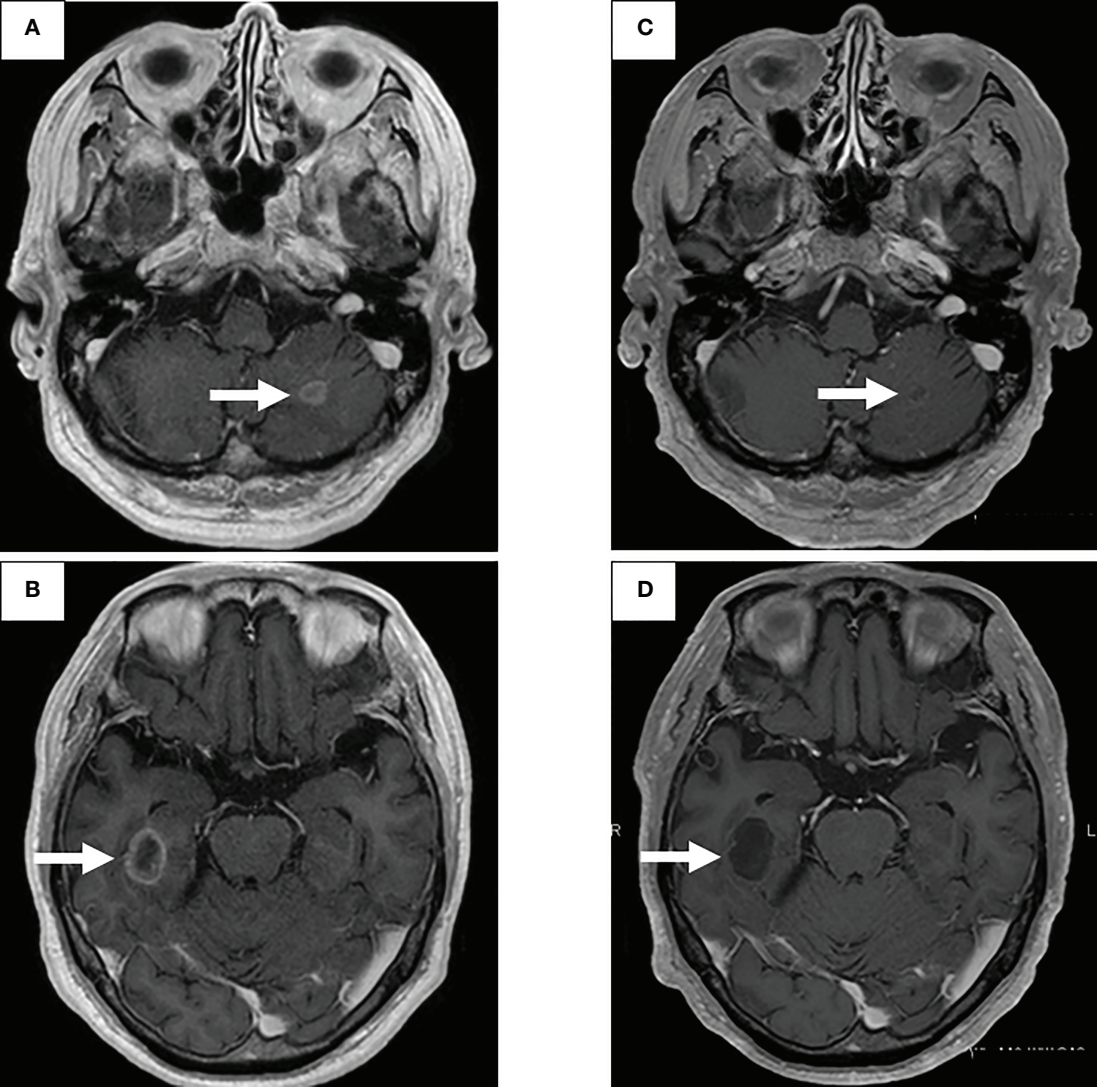
Therapeutic effect of brain metastasis using second-line treatment selpercatinib.

First-line treatment included carboplatin + pemetrexed + bevacizumab, and although a near complete response (CR) was obtained after 4 courses, nasal bleeding continued, and only pemetrexed was administered for maintenance therapy. CR was achieved over 25 courses of maintenance therapy (**Figure 1C**), but treatment was temporarily terminated due to a slight deterioration of renal function. One year post treatment, CT showed a slight progression in the primary lesion (**Figure 1D**), but the speed of regrowth was slow. One year after the progression was confirmed by CT (**Figure 1E**), pemetrexed monotherapy was resumed, and the primary lesion and lymph nodes decreased (**Figure 1F**). However, after 32 courses of maintenance therapy, a rapid systemic progression was noted. Due to the primary lesion regrowth (**Figure 1G**), contralateral lung metastasis, multiple liver metastases, a right adrenal metastasis, and multiple brain metastases (**Figures 2A, B**), adjustments to his treatment were necessary.

**Figure 3:**
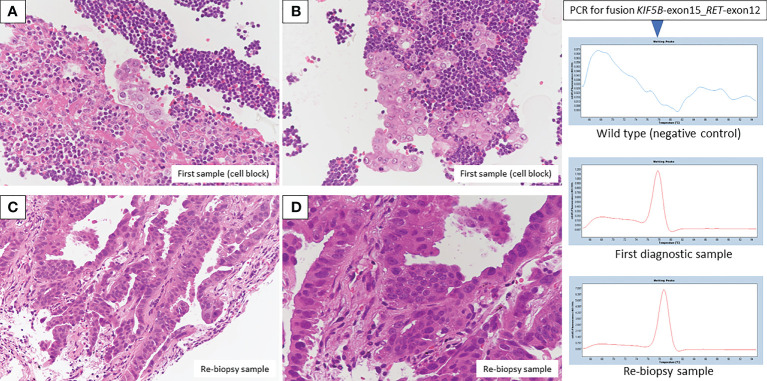
Pathologic findings from the first diagnostic cell block showed adenocarcinoma in the presence of many lymphocytes (**A, B**, H&E staining, original magnification × 400). A re-biopsy sample also showed adenocarcinoma with an alveolar, ductal structure (**C, D**, H&E staining, original magnification×200 and×400, respectively). Fusion *KIF5B*-exon 15; *RET*-exon 12 was detected by PCR/NGS assay from first diagnostic specimens, and NGS assay from re-biopsy specimens.

Bronchoscopic re-biopsy of the primary lesion revealed class V adenocarcinoma cytologically, and histological assessment confirmed this result. Oncomine™ Dx Target Test confirmed the patient was *RET* fusion gene-positive, and the RET inhibitor selpercatinib 240 mg was administered the following day. On day 13, CT revealed a good systemic response with all metastatic lesions, including brain metastases (**Figures 2C, D**), compared with baseline imaging (**Figure 1H**). Selpercatinib with a dose reduction (160 mg/day) for grade 2 elevated liver enzymes was continued. A high sensitivity next generation sequencing (NGS) panel system: lung cancer compact panel, with RNA assay using cytological brushing solution (6, 7), confirmed the fusion gene *KIF5B* exon 15; *RET* exon 12 (*K15RET12*). We could further confirm *RET* from formalin-fixed paraffin-embedded (FFPE) specimens from 7-year-old pleural effusion cell blocks (**Figures 3A, B**), which were morphologically similar to re-biopsy sample in terms of malignant cells with large nucleoli (**Figures 3C, D**). RNA (1256 ng) was collected from the initial cell block sample, with an RNA integrated number (RIN) value of 4.8. A *K15RET12* fusion peak was detected by single-plex polymerase chain reaction (PCR) (**Figure 3**) and NGS assay.

Finally, we provide additional information on PCR and NGS techniques. SYBR-Green real-time PCR assay with melting curve analysis was performed to detect RET fusion detection from archive cellblock sample. KOD SYBR^®^ qPCR/RT set (Toyobo) was used for reverse transcription and real time PCR. These assays were performed according to the manufacturer’s protocols. Non-tumor wild type sample was assayed in parallel as negative control. Peak call of 78 degree from melting curve chromatogram is the criteria for the detection of fusion positive. Moreover, sequence of fusion boundary was confirmed by NGS sequencing (Illumina Miseq 150 paired-end) of qPCR product.

Lung cancer compact panel assay was also performed for archive first diagnostic sample. *KIF5b-exon15_RET-exon12* fusion was also detected from this analysis. Moderate signal intensity of fusion variant was obtained from this assay (70 fusion boundary-positive reads out of 1,335 total sequencing reads). Lung cancer compact panel assay was performed according to the protocol described previously (8). Limit of detection of RET fusion detection by lung cancer compact panel is no more than 1% variant allele frequency.

## Discussion

Conventionally, gene mutations have been identified by the single-plex PCR method for individual gene mutations. The PCR, which has high sensitivity and specificity, a short turn-around time, and is relatively inexpensive, has become widespread mainly for the detection of EGFR. However, with the discovery of various lung cancer driver genes over the last five years, it is impractical to test single gene mutations sequentially due to time and sample consumption constraints. In 2017, the gene panel test, Oncomine Dx Target Test Multi-CDx system (Thermo Fisher Scientific, San Jose, CA, USA), which can simultaneously evaluate 46 cancer-related genes, was one of the first NGS panels for non-small cell lung cancer testing approved by the FDA (9). However, this batch test requires sufficient amounts of malignant cells in the collected tissue sample and qualified sample handling. Moreover, if a significant amount of time has passed since collection, the sample is often unsuitable for gene panel testing due to the deterioration of nucleic acid quality. Since the initial diagnosis in this case was made over 7 years ago, it was assumed that the nucleic acid quality would have deteriorated over that time. Therefore, re-biopsy of the primary lesion was performed.

This was a rare case where cytotoxic chemotherapy, and subsequent maintenance therapy, was remarkably effective for the primary lesion. Generally, progression-free survival for cytotoxic chemotherapy in advanced-stage gene mutation-positive lung adenocarcinoma is about 6 months (4, 5), and it is not often possible to continue treatment for more than one year. Even when treatment is continued beyond-progressive disease (beyond-PD), in most cases, treatment adjustments are unavoidable due to the systemic exacerbation of the disease. The prognosis for gene mutant lung adenocarcinoma without the use of molecular-targeted drugs is generally poor, but there are cases in which cancer development is observed over a very long period of time. In particular, *RET* fusion lung cancer has been reported to show a slower clinical course (10, 11). Hence, as in our patient, there are a number of cases where rare mutations have not been detected over long-term clinical courses.

Selpercatinib, similar to other molecular-targeted therapies, had sufficient systemic effects (12, 13), including brain metastases (14). In general, first-line treatment for gene mutation-positive lung adenocarcinoma is the corresponding molecular-targeted drug; however, this case suggests that the order of drugs used may be important when aiming for the longest overall survival by sequence therapy (15). In a phase 3 study of selpercatinib for *RET*-positive lung cancer, the efficacy of molecular-targeted drugs, in addition to the therapeutic effects of cytotoxic drugs will be clarified (16). It is noteworthy that we were able to detect *RET* fusion by extracting RNA from 7-year-old pleural effusion cell blocks, which contained a low percentage of malignant cells.

## Data availability statement

The original contributions presented in the study are included in the article/supplementary material. Further inquiries can be directed to the corresponding author.

## Ethics statement

Written informed consent was obtained from the patient for publication of this case report and any accompanying images. The research gene analysis was with ethics approval (HREC ID 4814).

## Author contributions

KM had full access to data in this case report and takes responsibility for the integrity and accuracy of data analysis. KM, HH, JU, HT and TI contributed to bronchoscopic examination and interpretation. NS, JK, SN and YS contributed to pathological evaluation and genetic analysis. KM and MM contributed to the scientific review and final approval of this manuscript. All authors contributed to the article and approved the submitted version.

## Acknowledgments

The authors thank Mr. Jason Tonge from St. Marianna University School of Medicine for the linguistic review of this manuscript.

## Conflict of interest

Author SN and YS was employed by DNA Chip Research Inc. The research gene analysis of this case was conducted by DNA Chip Research Inc., Tokyo, Japan.

The remaining authors declare that the research was conducted in the absence of any commercial or financial relationships that could be construed as a potential conflict of interest.

## Publisher’s note

All claims expressed in this article are solely those of the authors and do not necessarily represent those of their affiliated organizations, or those of the publisher, the editors and the reviewers. Any product that may be evaluated in this article, or claim that may be made by its manufacturer, is not guaranteed or endorsed by the publisher.

## References

[1] Mark GK Bruce EJ Lynne DB Kwiatkowski DJ Iafrate AJ Wistuba II. Using multiplexed assays of oncogenic drivers in lung cancers to select targeted drugs. JAMA (2014) 311(19):1998–2006. doi: 10.1001/jama.2014.3741 24846037 PMC4163053

[2] Gambardella V Tarazona N Cejalvo JM Lombardi P Huerta M Roselló S. Personalized medicine: Recent progress in cancer therapy. Cancers (2020) 12(4):1009. doi: 10.3390/cancers12041009 32325878 PMC7226371

[3] Mosele F Remon J Mateo J Westphalen CB Barlesi F Lolkema MP. Recommendations for the use of next-generation sequencing (NGS) for patients with metastatic cancers: a report from the ESMO precision medicine working group. Ann Oncol (2020) 31(11):1491–505. doi: 10.1016/j.annonc.2020.07.014 32853681

[4] Soria JC Tan DSW Chiari R Wu YL Paz-Ares L Wolf J. First-line ceritinib versus platinum-based chemotherapy in advanced ALK-rearranged non-small-cell lung cancer (ASCEND-4): a randomised, open-label, phase 3 study. Lancet (2017) 389(10072):917–29. doi: 10.1016/S0140-6736(17)30123-X 28126333

[5] Rosell R Carcereny E Gervais R Vergnenegre A Massuti B Felip E. Erlotinib versus standard chemotherapy as first-line treatment for European patients with advanced EGFR mutation-positive non-small-cell lung cancer (EURTAC): a multicentre, open-label, randomised phase 3 trial. Lancet Oncol (2012) 13(3):239–46. doi: 10.1016/S1470-2045(11)70393-X 22285168

[6] Morikawa K Kida H Handa H Inoue T Saji H Koike J. A prospective validation study of lung cancer gene panel testing using cytological specimens. Cancers (2022) 14(15):3784. doi: 10.3390/cancers14153784 35954448 PMC9367394

[7] Morikawa K Kinoshita K Kida H Inoue T Mineshita M. Preliminary results of NGS gene panel test using NSCLC sputum cytology and therapeutic effect using corresponding molecular-targeted drugs. Genes (2022) 13(5):812. 35627198 10.3390/genes13050812 PMC9141607

[8] Kato K Okami J Nakamura H Honma K Sato Y Nakamura S. Analytical performance of a highly sensitive system to detect gene variants using next-generation sequencing for lung cancer companion diagnostics. medRxiv (2021). doi: 10.1101/2021.10.13.21264976 PMC10137435 37189577

[9] FDA U. Summary of safety and effectiveness data for oncomine dx target test (2017). Available at: https://www.accessdata.fda.gov/cdrh_docs/pdf16/P160045B.pdf (Accessed 27 March 2022).

[10] Drilon A Bergagnini I Delasos L Sabari J Woo KM Plodkowski A. Clinical outcomes with pemetrexed-based systemic therapies in RET-rearranged lung cancers. Ann Oncol (2016) 27(7):1286–91. doi: 10.1093/annonc/mdw163 PMC4922319 27056998

[11] Shen T Pu X Wang L Yu Z Li J Zhang Y. Association between RET fusions and efficacy of pemetrexed-based chemotherapy for patients with advanced NSCLC in China: A multicenter retrospective study. Clin Lung Cancer (2020) 21(5):e349–54. doi: 10.1016/j.cllc.2020.02.006 32143967

[12] Illini O Hochmair MJ Fabikan H Weinlinger C Tufman A Swalduz A. Selpercatinib in RET fusion-positive non-small-cell lung cancer (SIREN): a retrospective analysis of patients treated through an access program. Ther Adv Med Oncol (2021) 13:17588359211019675. doi: 10.1177/17588359211019675 34178121 PMC8202258

[13] Hess LM Han Y Zhu YE Bhandari NR Sireci A. Characteristics and outcomes of patients with RET-fusion positive non-small lung cancer in real-world practice in the united states. BMC Cancer (2021) 21(1):28. doi: 10.1186/s12885-020-07714-3 33402119 PMC7786962

[14] Subbiah V Gainor JF Oxnard GR Tan DSW Owen DH Cho BC. Intracranial efficacy of selpercatinib in RET fusion-positive non-small cell lung cancers on the LIBRETTO-001 trial. Clin Cancer Res (2021) 27(15):4160–7. doi: 10.1158/1078-0432.CCR-21-0800 PMC8447251 34088726

[15] Knetki-Wróblewska M Wojas-Krawczyk K Kowalski DM Krzakowski M. Non-Small-Cell lung cancer patients with coexistence of high PD-L1 expression and RET fusion-which path should we follow? case reports and literature review. J Clin Med (2022) 11(6):1630. doi: 10.3390/jcm11061630 35329956 PMC8949444

[16] Solomon BJ Zhou CC Drilon A Park K Wolf J Elamin Y. Phase III study of selpercatinib versus chemotherapy ± pembrolizumab in untreated RET positive non-small-cell lung cancer. Future Oncol (2021) 17(7):763–73. doi: 10.2217/fon-2020-0935 33150799

